# Clinical determinants impacting overall survival of patients with operable brain metastases from non-small cell lung cancer

**DOI:** 10.3389/fonc.2022.951805

**Published:** 2022-10-20

**Authors:** Andras Piffko, Benedikt Asey, Lasse Dührsen, Inka Ristow, Johannes Salamon, Harriet Wikman, Cecile L. Maire, Katrin Lamszus, Manfred Westphal, Thomas Sauvigny, Malte Mohme

**Affiliations:** ^1^ Department of Neurosurgery, University Medical Center Hamburg-Eppendorf, Hamburg, Germany; ^2^ Department of Radiation and Cellular Oncology, University of Chicago, Chicago, IL, United States; ^3^ The Ludwig Center for Metastasis Research, University of Chicago, Chicago, IL, United States; ^4^ Department of Diagnostic and Interventional Radiology and Nuclear Medicine, University Medical Center Hamburg-Eppendorf, Hamburg, Germany; ^5^ Department of Tumor Biology, University Medical Center Hamburg-Eppendorf, Hamburg, Germany

**Keywords:** NSCLC, metastasis, brain metastasis, survival, resectable, surgery

## Abstract

Non-small cell lung cancer (NSCLC) is currently the leading cause of cancer-related death worldwide, and the incidence of brain metastases (BM) in NSCLC patients is continuously increasing. The recent improvements of systemic treatment in NSCLC necessitate continuous updates on prognostic subgroups and factors determining overall survival (OS). In order to improve clinical decision-making in tumor boards, we investigated the clinical determinants affecting survival in patients with resectable NSCLC BM. A retrospective analysis was conducted of NSCLC patients with surgically resectable BM treated in our institution between 01/2015 and 12/2020. The relevant clinical factors affecting survival identified by univariate analysis were included in a multivariate logistic regression model. Overall, 264 patients were identified, with a mean age of 62.39 ± 9.98 years at the initial diagnosis of NSCLC BM and OS of 23.22 ± 1.71 months. The factors that significantly affected OS from the time of primary tumor diagnosis included the systemic metastatic load (median: 28.40 ± 4.82 vs. 40.93 ± 11.18 months, p = 0.021) as well as a number of BM <2 (median: 17.20 ± 2.52 vs. 32.53 ± 3.35 months, p = 0.014). When adjusted for survival time after neurosurgical intervention, a significant survival benefit was found in patients <60 years (median 16.13 ± 3.85 vs. 9.20 ± 1.39 months, p = 0.011) and, among others, patients without any concurrent systemic metastases at time of NSCLC BM diagnosis. Our data shows that the number of BM (singular/solitary), the Karnofsky Performance Status, gender, and age but not localization (infra-/supratentorial), mass-edema index or time to BM occurrence impact OS, and postsurgical survival in NSCLC BM patients. Additionally, our study shows that patients in prognostically favorable clinical subgroups an OS, which differs significantly from current statements in literature. The described clinically relevant factors may improve the understanding of the risks and the course of this disease and Faid future clinical decision making in tumor boards.

## Introduction

Non–small cell lung cancer (NSCLC) is the leading cause of death in cancer patients (1.3 million/year) worldwide, accounting for 25% of all cancer-related deaths (1). Despite significant improvements in treatment, especially within the field of immuno-oncology (2–4), NSCLC mortality remains extremely high and the overall 5-year survival rates rarely exceed 15% (1, 5). Approximately 40% of patients with stage III NSCLC will develop brain metastases (BM) (6). The incidence of brain metastases continues to rise, partly as a result of improved extracranial disease control and subsequently prolonged survival (7, 8), partly due to other factors, such as more readily available and increasingly accurate diagnostic procedures, which facilitate an earlier and a more frequent diagnosis of intracranial disease. In clinical practice, the occurrence of BM at the initial diagnosis (ID) or during the treatment course of NSCLC has been associated with a reduction of the quality of life, and, more importantly, with a dismal disease course and poor prognosis. In addition, BM may lead to neurological impairments by affecting both cognitive and sensory functions and thus further diminish the quality of life (9, 10). However, due to the high degree of heterogeneity in metastatic dissemination, the timing of BM occurrence, and various clinical determinants, such as gender, age, systemic tumor dissemination, and clinical factors that impact overall survival (OS), reliable data on the differences in the disease course for patients undergoing neurosurgical resection are scarce. To improve future treatment strategies and tumor board decision-making processes, a better understanding of the risk stratification for patients with NSCLC BM patients is urgently needed. Therefore, the aim of our study was to analyze clinical determinants affecting patient survival after ID, as well as survival after neurosurgical resection.

## Results

### Study cohort

We identified 264 patients who were treated for brain metastatic NSCLC in our institution between 01/2015 and 12/2020. The mean age at the ID of NSCLC was 61.54 ± 10.06 years (range 33–83 years). The male-to-female ratio was 1:1.18. The median time to BM development was 10.98 ± 20.62 months, thus accounting for the mean age at the neurosurgical intervention of 62.39 ± 9.98 years. In total, 61.38% (n=151/246) of patients were diagnosed with synchronous NSCLC BM at our institution without a prior NSCLC diagnosis and were thus termed “BM at ID.” Of these 151 patients, 81 (53.64%) showed an additional synchronous metastatic disease of other organs. The average number of brain metastases was 1.93 ± 0.136, and the mean size of the largest observed BM lesion was 12.93 ± 1.51 cm^3^. The majority of the patients primarily underwent surgery with the goal of total resection [96.06% (n = 244/256)]. Partial resection was performed in 1.56% (n = 4/256) of cases and tissue biopsies in 3.12% (n = 8/256). The median OS from the time of the primary tumor diagnosis was 15.00 ± 2.27 months. Postoperative complications affected 26/264 patients (9.85%). A total of 10 (3.78%) complications included postoperative hemorrhages at the resection site, three (1.13%) patients suffered from postoperative CSF fistulas, eight (3.03%) received antibiotics for postoperative wound infections, four (1.51%) developed hydrocephalus, and postoperative cerebral infarctions were found in one (0.38%) patient with surgical complications.

The histological subtype classification of the BM tissue was available in 84.09% (n=222/264) of cases. The most common NSCLC histological diagnoses based on the analysis of the BM tissue were comprised of adenocarcinomas (n=183/222, 82.43%) followed by squamous cell (n=21/222, 9.46%) and neuroendocrine carcinomas (n=11/222, 4.95%) and not-otherwise-specified (NOS) histology (n=7/222, 3.15%). A single intracerebral metastasis was observed in two-thirds of patients (67.4%, n=159/236), while 11.9% (n=28/236) and 20.8% of patients (n=49/236) patients presented with two or three and more intracerebral tumor manifestations, respectively. Information about the mutation status of the primary NSCLC was available in n=66/264 cases (25.0%). The most commonly observed driver mutations of the primary tumor affected *TP53* [n=9/66 (13.64%)] and *KRAS* [n=9/66 (13.64%)], followed by *EGFR* [n=7/36 (10.61%)]. Programmed cell death 1 ligand 1 (PD-L1) expression was analyzed in 44/264 (16.67%) of primary tumor samples, and the mean PD-L1 expression was graded 45.35% in tumor cells (range 0%–90%) and 3.26% on infiltrating immune cells (range 0%–20%).

The mutational analysis information of the BM tissue was available in n=92/264 (34.85%) of all cases in the analyzed time period. The most observed driver mutation, similar to our observation in the primary tumor, affected *TP53*, detected in 25.0% (n=23/92) of cases, followed by *KRAS* (16.30%, n=15/92) and *EGFR* (9.78%, n=9/92). Other druggable mutations such as *ALK* [n=1/92 (2.78%)] and *ROS* [n=2/92 (2.78%)] were rare in the observed patient cohort. PD-L1 expression in the BM tissue was analyzed in n=74/264 (28.03%) of cases, and the mean PD-L1 expression was graded 36.88% in tumor cells (range 0%–90%) and 4.01% on infiltrating immune cells (range 0%–20%)

In total, 48.5% of patients (n=128/264) received no NSCLC-specific treatment before the neurosurgical intervention [n=23/151, (15.2%) patients within the “BM at ID” group had been diagnosed with NSCLC less than 4 weeks before the identification of brain metastases and had thus just begun first treatment chemotherapy cycles]. Information about preoperative adjuvant treatments was available in 159/264 patients (60.22%). Of these patients, n=133/159 (83.65%) received chemotherapy (CTX). As expected, the most commonly prescribed chemotherapeutics—applied in n=126/133 cases (94.74%)—were platinum based (containing either cisplatinum or carboplatinum). Information about the postoperative radiotherapy of BM was available in n=159/264 (60.23%) of cases. Out of 159 cases, 45 (28.30%) received whole-brain radiotherapy (WBRT) and n=8/159 (5.03%) received no postoperative radiation treatment, while the remaining 109 cases underwent fractionated stereotactic brain radiotherapy (SBRT) or gamma knife radio surgery (GKRS), with the additional treatment of non-resected lesions in cases deemed necessary. The most commonly applied cumulative dose in SBRT was 35 Gy in seven fractions (25/109 cases), while the most commonly applied fractionation regiment in WBRT consisted of 10 × 3 Gy [30 Gy cumulative dose, 23/45 cases (51.11%)].

We scored the patient cohort according to the Karnofsky Performance Status (KPS) and included the values in our analyses at three distinct points in time: 1) pre-operative (mean 76.25 ± 16.65) 2) postoperative (mean 80.85 ± 18.33), and 3) the last documented score available (mean 29.15 ± 38.39) (**Table 1**). Further, detailed clinical information is displayed in **Table 1**.

**Table 1:** Clinical information of patient cohort.

	N (%)	Mean	Std. dev.
**Age at ID**	**255/264 (96.60)**	61.54	10.06
**Age at surgery**	**258/264 (94.73)**	62.39	9.98
**Female gender**	**143/264 (54.17)**		
**Time to BM development (months)**	**246/264 (93.18)**	10.98	20.62
**BM at ID**	**151/264 (61.38)**		
**KPS pre-op**	**253/264 (95.83**)****	76.25	16.65
**KPS post-op**	**246/264 (93.18)**	80.85	18.33
**KPS last documented**	**235/264 (89.02)**	29.15	38.39
**Histology**	**222/264 (84.09)**		
Adeno	183/222 (82.43)		
Squamous cell	21/222 (9.46)		
Neuro-endocrine	11/222 (4.95)		
NOS	7/222 (3.15)		
**Initial T status**	**201/264 (76.14)**		
T1	47/201 (23.38)		
T2	59/201 (29.35)		
T3	41/201 (20.40)		
T4	55/201 (27.36)		
**Initial N status**	**200/264 (75.75)**		
N0	69/200 (34.50)		
N1	31/200 (15.50)		
N2	56/200 (28.00)		
N3	45/200 (27.50)		
**Initial M status**	**224/264 (94.95)**		
M0	61/264 (27.23)		
M1	164/264 (73.21)		
**Mets at NSCLC ID (other than BM)**	**100/264 (37.88)**		
Liver	12/100 (12.00)		
Lung	29/100 (29.00)		
Bone	22/100 (22.00)		
Adrenal Gland	29/100 (29.00)		
Other	8/100 (8.00)		
**BM count**	**236/264 (89.39)**		
1	159/236 (67.37)		
2	28/236 (11.86)		
≥3	49/236 (20.76)		
**BM localization (largest lesion)**	**225/264 (85.22)**		
Supratentorial	181/225 (80.44)		
Infratentorial	44/225 (19.56)		
**Primary tumor mutational status**	**66/264 (25.00)**		
KRAS	9/66 (13.64)		
EGFR	7/66 (10.61)		
MET	3/66 (4.55)		
BRAF	0/66 (0)		
ALK	1/66 (1.52)		
ROS	1/66 (1.52)		
FGFR3	4/66 (6.06)		
PIK3CA	2/66 (3.03)		
TP53	9/66 (13.64)		
**BM mutational status**	**92/264 (34.85)**		
KRAS	15/92 (16.30)		
EGFR	9/92 (9.78)		
MET	1/92 (1.09)		
BRAF	2/92 (2.17)		
ALK	1/92 (1.09)		
ROS	2/92 (2.17)		
FGFR3	2/92 (2.17)		
PIKC3A	3/92 (3.26)		
TP53	23/92 (25.00)		
**Treatment after NSCLC diagnosis**	**159/264 (60.23)**		
**CTX after NSCLC diagnosis**	**133/264 (83.65)**		
**RT after BM diagnosis**	**159/264 (60.23)**		
WBRT	8/159 (5.03)		
SBRT / GKS	109/159 (71.24)		
**ICB**	**46/264 (17.42)**		
**Type of operative approach**	**256/264 (97.00)**		
Total resection	244/256 (96.06)		
Partial resection	4/256 (1.56)		
Biopsy	8/256 (3.12)		
**Known positive smoking status**	**133/264 (50.37)**		
**Seizures**	**67/264 (25.38)**		
**Meningeosis carcinomatosa**	**8/264 (3.03)**		
**Follow up time**	**261/264 (98.86)**	22.56	26.86

KPS, Karnofsky Performance Status; NOS, not otherwise specified; NSCLC, non-small cell lung cancer; ID, initial diagnosis; BM, brain metastasis; CTX, chemotherapy; RT, radiation therapy; WBRT, whole brain radiotherapy; SBRT, stereotactic body radiation; GKS, gamma knife surgery; ICB, immune checkpoint blockade.

### Clinical determinants for overall survival

**Figure 1:**
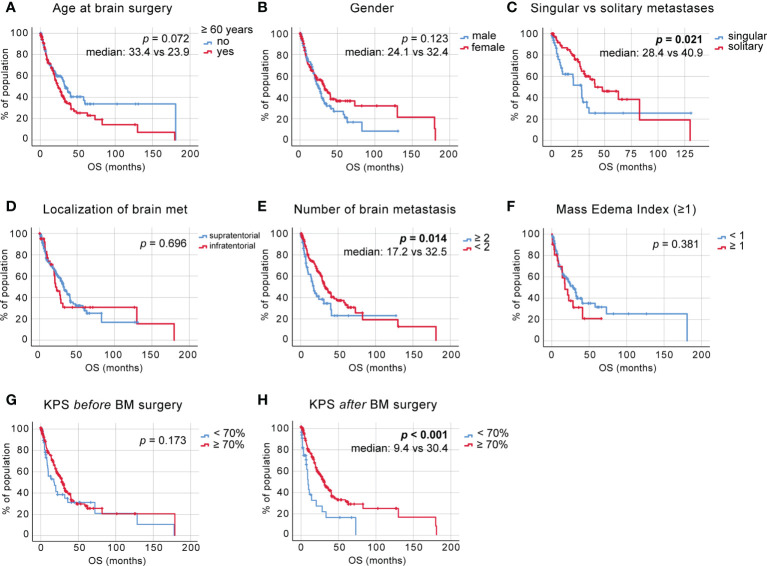
Overall survival (OS) in non-small cell lung cancer (NSCLC) brain metastases (BM) patients **(A)** Kaplan–Meier survival analysis of OS from the time point of initial diagnosis in patients <60 and >60 years of age, **(B)** in male vs. female patients, **(C)** in patients with a singular vs. solitary BM status, **(D)** depending on BM localization, **(E)** depending on the number of BM, **(F)** depending on the mass-edema index (MEI), **(G)** depending on the preoperative Karnofsky Performance Status (KPS) score, and **(H)** depending on the KPS score at discharge.

Overall, 97 of 255 patients (38.04%) were younger than 60 years at the time of neurosurgical intervention. The comparison of OS between patients aged over versus under 60 years (**Figure 1A**) indicated a survival benefit of younger patients without quite reaching statistical significance in this cohort **[**p = 0.072, log-rank (Mantel–Cox) test**]**. A similar trend of a potential survival benefit was observed with female sex (n=133/251, 52.98%); however, as above, the difference did not prove statistically significant **[** **Figure 1B**, p = 0.123, log-rank (Mantel–Cox) test**]**. The systemic metastatic load at time of initial BM diagnosis was evaluated by comparing a singular BM status (one BM lesion, with concurrent systemic metastases) to a solitary BM status (one BM lesion, without further systemic metastases). In total, 67% (n=68/120) of patients presented with solitary BM status. As expected, the lack of additional systemic metastases in solitary BM patients correlated with a significant survival benefit when contrasted with the singular BM group (**Figure 1C**, median: 28.40 ± 4.82 vs. 40.93 ± 11.18 months respectively, p = 0.021, log-rank (Mantel–Cox) test). We dichotomized based on the supra-/infratentorial localization of the singular BM lesion (or localization of the largest lesion in case of multiple BM); however, we did not observe a significant effect on OS **[** **Figure 1D**, p = 0.696, log-rank (Mantel–Cox) test**]**. The total number of BM did, however, significantly affect the OS of the patient cohort, benefitting patients affected by <2 BM at the time of diagnosis, irrespective of the occurrence of additional systemic metastases [**Figure 1E**, median: 17.20 ± 2.52 vs. 32.53 ± 3.35 months, p = 0.014, log-rank (Mantel–Cox) test].

The mass-edema index (MEI), calculated as size of contrast-enhanced area in T1-weighted MRI/the size of peritumoral brain edema (PTB) in T2/flair-weighted MRI, did not affect OS [**Figure 1F**, p = 0.381, log-rank (Mantel–Cox) test].

The cut-off value for high KPS was set at 70%. This analysis showed that the preoperatively high KPS scores do not confer a significant survival benefit [**Figure 1G**, p = 0.173, log-rank (Mantel–Cox) test]; however, a postoperatively scored KPS of 70% or higher does [**Figure 1H**, median 9.47 ± 0.94, vs. 30.43 ± 2.76, p < 0.001, log-rank (Mantel–Cox) test].

### Clinical determinants for survival time after brain surgery

**Figure 2:**
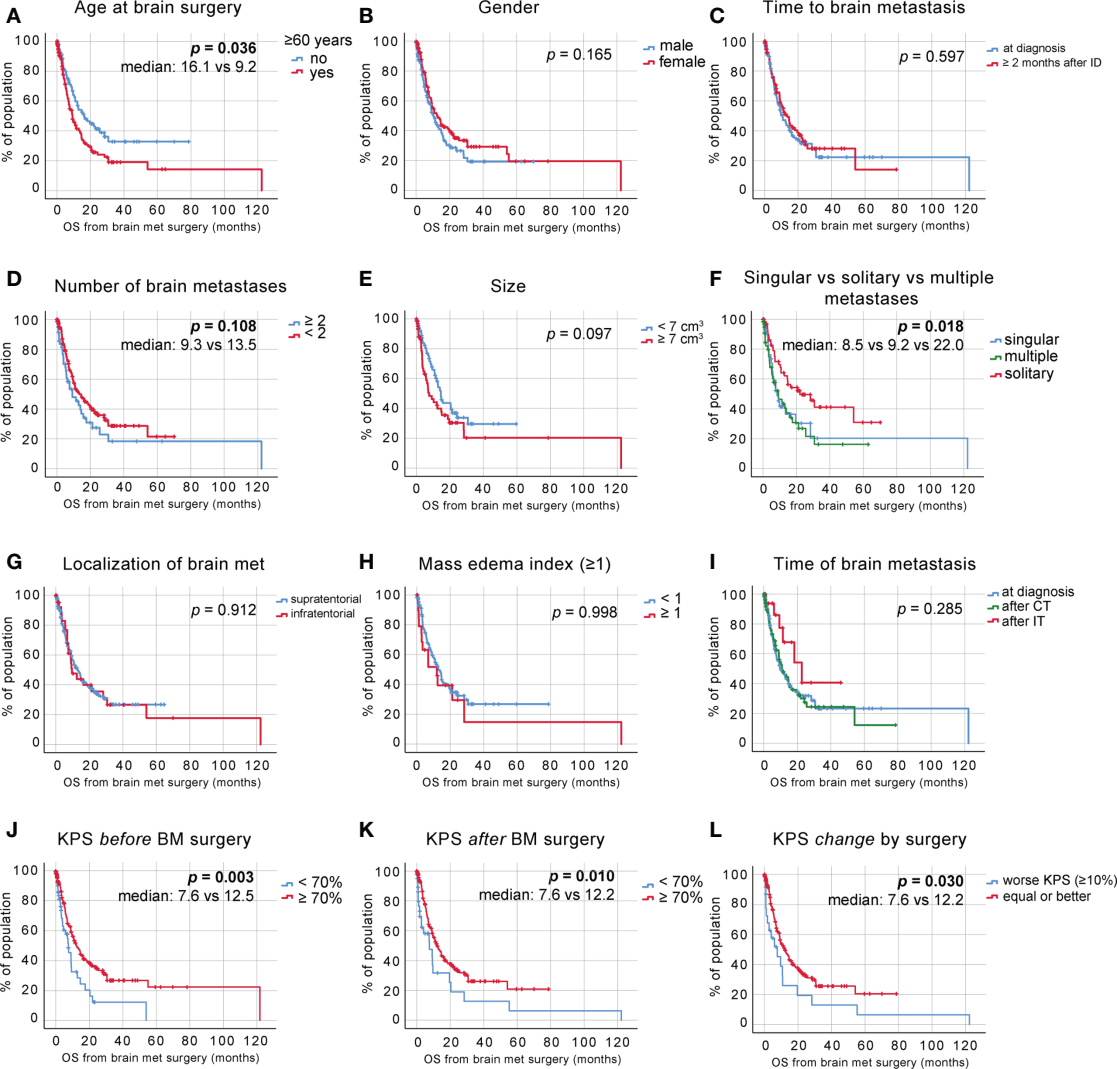
OS postneurosurgical intervention. **(A)** Kaplan–Meier survival analysis postneurosurgical intervention in patients <60 and >60 years of age, **(B)** in male vs. female patients, **(C)** depending on the time to BM diagnosis, **(D)** depending on the number of BM, **(E)** depending on the size of BM, **(F)** in patients with singular vs. solitary vs. multiple BM status, **(G)** depending on BM localization, **(H)** depending on the MEI, **(I)** depending on postdiagnosis treatment (at diagnosis—no treatment pre-non-small cell lung cancer (NSCLC) BM diagnosis, after CT—chemotherapy treatment pre-NSCLC BM diagnosis, and after immunotherapy (IT)— immune checkpoint blockade treatment pre-NSCLC BM diagnosis), **(J)** depending on the preoperative KPS score, **(K)** depending on the KPS score at discharge, and **(L)** depending on the KPS score change due to surgical intervention.

We adjusted for the duration of survival after neurosurgical intervention and observed a significance in the previously suggested survival benefit of patients aged 60 years or younger [**Figure 2A**, median 16.13 ± 3.85 vs. 9.20 ± 1.39 months, p = 0.036, log-rank (Mantel–Cox) test]. The adjusted OS was 11.47 ± 0.95 months. A statistically significant difference between survival rates after brain surgery was again not reached between male and female patients, with a trend pointing toward a survival benefit of female patients [**Figure 2B**, p = 0.165, log-rank (Mantel–Cox) test]. In addition, no survival benefit was seen in patients diagnosed with BM less than 2 months after the NSCLC diagnosis [**Figure 2C**, p = 0.597, log-rank (Mantel–Cox) test].

Despite the observed statistically significant OS benefit of patients affected with fewer than two BM, we did not find the same effect on the survival time after neurosurgical intervention [**Figure 2D**, p = 0.108, log-rank (Mantel–Cox) test]. When comparing the size of solitary BM as seen in the volumetric measurements of contrast-enhanced areas in T1-weighted magnetic resonance imaging (MRI), we observed a trend toward an improved survival of patients with tumors <7 cm^3^ [**Figure 2E**, p = 0.097, log-rank (Mantel–Cox) test].

To further delineate the effects of the systemic metastatic status on OS, patients were stratified according to their systemic and intracranial metastatic load at the time of BM diagnosis. We stratified the patients into three groups; singular brain metastasis with concurrent systemic metastases, solitary brain metastasis (no concurrent systemic metastases), and multiple brainmetastases and observed a significant survival benefit inpatients with solitary BM status [**Figure 2F**, median: 8.47 ± 1.71, 22.03 ± 7.29 and 9.20 ± 2.81 months, respectively, p=0.018, log-rank (Mantel–Cox) test].

A supra- vs. infratentorial localization of BM had no effect on survival again after neurosurgical [**Figure 2G**, p = 0.912, log-rank (Mantel–Cox) test], neither did the comparison between mass-edema indices <1 and >1 [**Figure 2H**, p = 0.998, log-rank (Mantel–Cox) test].

We compared the groups of patients diagnosed with BM at NSCLC diagnosis, patients who previously received systemic chemotherapy (CT) for their underlying NSCLC disease (labeled “after CT”), and patients who received any combination of immune checkpoint blockade (ICB) and chemotherapeutics prior to their BM diagnosis (labeled “after IT”) and saw a slight trend toward a survival benefit of patients that previously received a combination of CT and ICB [**Figure 2I**, median 9.80 ± 1.87 vs. 22.73 ± 7.09 vs. 10.87 ± 2.34 months, respectively, p = 0.285, log-rank (Mantel–Cox) test], which we will follow up in further studies.

We again scored our patients according to the KPS and analyzed survival post-BM resection. This analysis showed that patients with a KPS of >70 at initial diagnosis show significantly improved postoperative survival [**Figure 2J**, median 7.57 ± 1.66 vs. 12.5 ± 1.57 months, p = 0.003, log-rank (Mantel–Cox) test]. The increase in survival also became apparent when comparing KPS scores at discharge [**Figure 2K**, median 7.60 ± 3.24 vs. 12.23 ± 1.63 months, p = 0.010, log-rank (Mantel–Cox) test]. In addition, KPS changes due to the surgical intervention demonstrated to also have a significant impact on survival from the time point of BM surgery [**Figure 2L**, median 7.60 ± 3.41 and 12.23 ± 1.96, respectively, p = 0.030].

### Clinically favorable patient population

**Figure 3:**
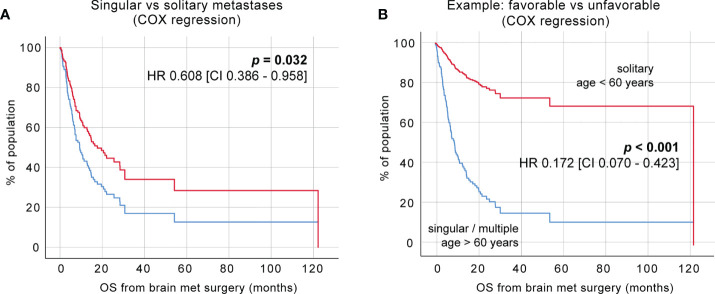
Identification of favorable clinical subgroups. **(A)** Cox regression of a singular vs. solitary BM status. **(B)** Cox regression of favorable vs. unfavorable patient groups.

To further dissect the effects of these clinical determinants on the survival probabilities of specific patient groups after surgical intervention, we incorporated relevant findings from the univariate analyses into a multivariate analysis. Significant factors affecting postoperative survival are shown in **Table 2** and were incorporated in a Cox regression analysis. This includes 1) the presence of solitary vs. multiple BM (HR 1.034, 95% CI 0.316 – 0.819, p = 0.005) and 2) pre-operative KPS (HR 0.981, 95% CI 0.967 – 0.996, p = 0.011) as well as age (HR 1.034, 95% CI 1.009 – 1.059, p = 0.007). Stratification of the patient cohort by singular or solitary BM status showed a significant survival benefit of patients with solitary BM in Cox regression analysis [**Figure 3A**, HR =0.608, CI 0.386 - 0.958, p = 0.032].

**Table 2:** Clinical determinants for overall survival and survival after surgery.

Clinical determinants for overall survival	Univariate (Log Rank) p-value	COX regression HR, 95%CI, p-value
Age (<60 years)	*p* = 0.072	
Female gender	*p* = 0.123	
Solitary BM status	*p* = 0.021*	0.509, 0.316 – 0.819, p = 0.005
BM localization	*p* = 0.696	
Mass-edema index	*p* = 0.381	
KPS pre-op	*p* = 0.173	
KPS post-op	p < 0.001*	0.980, 0.968 – 0.992, p = 0.001
**Clinical determinants for survival after surgery**		
Age (<60 years)	*p* = 0.036*	1.034, 1.009 – 1.059, p = 0.007
Female gender	*p* = 0.165	
BM at NSCLC ID	*p* = 0.597	
<2 BM	*p* = 0.108	
Size (<7cm^3^)	*p* = 0.097	
Solitary BM status	*p* = 0.032*	1.034, 0.316 – 0.819, p = 0.005
BM localization	*p* = 0.912	
Mass-edema index	*p* = 0.998	
Previous treatment	*p* = 0.285	
KPS pre-op	*p* = 0.003*	0.981, 0.967 – 0.996, p = 0.011

*included in COX regression.

BM, brain metastases; KPS, Karnofsky Performance Status; NSCLC, non-small cell lung cancer; ID, initial diagnosis.

Further, we classified the patient cohort into a favorable outcome group (solitary BM, age <60 years) and an unfavorable outcome group (singular and multiple BM, age >60 years) and performed Cox regression analysis, which demonstrated significantly increased survival after BM surgery in patients aged 60 years and younger with a solitary BM status (no concurrent systemic metastases) [**Figure 3B**, HR 0.172, CI 0.070 – 0.423, p <0.001].

## Discussion

In this large single-center retrospective analysis of NSCLC BM patients, we aimed to understand the effects of the most common clinical determinants on patient survival after the initial diagnosis and neurosurgical intervention. The study cohort contained 264 patients, with similar clinical characteristics as had previously been reported for the patients affected by this disease (11–13). The median OS of the herein-reported patient cohort was 15.0 ± 2.27 months, which was thus higher than the 377 retrospectively analyzed patients by Jünger et al. (median OS 14.1 months, 95% CI 12.2 – 15.8) (12), or 126 NSCLC patients analyzed by Fabi et al. (median OS 12 months, CI 9.0 – 16.0) (13). The reasons for this apparent increase in OS are manifold and may include, among others, improvements in surgical and imaging techniques and targeted molecular therapies as well as recent technological developments in radiotherapy. Intriguingly, the presented cohort consisted of 54.17% female patients, despite a distinctively higher prevalence of NSCLC diagnoses in male patients within the German population [for example, 34.690/53.500 (64.84%) of NSCLC patients in 2016 were men (14)]. Accordingly, most studies with comparable patient populations have reported a higher incidence of the male gender [54.9% in the study by Jünger and colleagues (12) and 52.4% in the study by Smith et al. (15)]. A possible explanation was sought in the predominance of adenocarcinomas identified in our patient cohort (82.43%) as these are generally more commonly found—and steadily increasing—in female patients (14); however, similar disseminations of histological diagnoses could be observed in the aforementioned studies [78.4% and 82.0%, respectively, (12, 15)]. Thus, additional factors might have contributed to the predominance of female patients that will be interesting to evaluate in future studies.

The rise of SBRT as first-line postoperative treatment modality has enabled the localized treatment of multiple intracerebral lesions and, partly owing to concerns about cognitive decline in patients receiving WBRT, has been recommended for patients with one-to-four lesions in the American Society of Radiation Oncology (ASTRO) guidelines since 2012 (16). More recently, further technological improvements have enabled the expansion of stereotactic radiosurgery (SRS) indications to include patients with up to 10 BM lesions and multiple clinical trials exploring the efficacy of SRS in patients with >20 BM are currently ongoing (17, 18), thus enabling a more targeted and localized control of brain metastatic disease for an increasing number of patients. Moreover, despite suffering from inconsistent response rates in cerebral metastases, recent targeted therapies such as ICB have undoubtedly enabled a more personalized treatment approach in oncological patients. In our analysis, we saw that a treatment with a combination of chemotherapeutics and ICB seemed to favor longer survival without quite reaching statistical significance and thus has to be analyzed in a larger cohort in the near future. A recent study by Rounis etal. (19) focused specifically on a subgroup of patients who received PD-1/PD-L1 inhibitors as treatment for NSCLC BM and found that specific clinical parameters, such as age <70 years, prior CNS radiation, and the synchronous appearance of BM, significantly affected ICB disease control. However, it is important to point out that this study was focused on the patients who received ICB as monotherapy, as opposed to the patients included in our cohort, who received any combination of CT and ICB.

Our findings suggest that the most significant factors affecting OS are 1) a lack of additional systemic metastases (“solitary BM lesion”) and 2) the number of BM lesions at the time of BM diagnosis. The stratification of patients into singular BM with concurrent systemic metastases, solitary BM without systemic affection, or multiple metastases at the time of BM surgery has shown a significant difference with an almost threefold increase in survival post neurosurgical intervention in patients with a solitary BM status (median 8.5 vs. 9.2 vs. 22.0 months, respectively, **Figure 2F**). This discernible effect of the number of metastatic lesions on OS underlines the idea that oligometastatic disease—as proposed by Samuel Hellman and Ralph Weichselbaum in their seminal paper in 1995 (20)—might represent a different spectrum of metastatic disease than widespread disease and should, in this case, be considered amenable to a curative therapeutic strategy (20, 21). The paradigm shift necessary to distinguish oligometastasized patients from those with widespread, multifocal disease could help identify clinically favorable subgroups and enhance our understanding of personalized treatment strategies. Purely by focusing on factors positively correlated with OS, we were able to identify patients with clinically favorable features (solitary BM, age <60 years) with a mean OS of 53.82 months, which is noteworthy since it extends the scope of patient survival far beyond the mean values currently discussed for BM patients in the literature. Nevertheless, more focused studies are needed to identify these patient groups and understand the nature and extent of cerebral oligometastatic disease. A positive outlook is provided by the studies of oligometastatic disease affecting other organs, such as a recent study by Pitroda etal. (22), in which the authors performed an integrative analysis of 134 patients affected by one-to-three liver metastases and were able to identify three groups with a 10-year OS rate of 94%, 45%, and 19%, respectively (22).

Additionally, to the number of metastases, when adjusted for survival after neurosurgical intervention, we identified age <60 years as a predictor of significantly increased patient survival after analyzing multiple age cut-off values. Interestingly, this survival difference only delineated as a significant predictor after adjusting the survival for values after neurosurgical intervention as opposed to the survival time after initial BM diagnosis. Few of the aforementioned studies adjusted for this distinction; thus, it would be interesting to evaluate whether and how the survival benefit perceived in younger patients is connected with neurosurgical interventions. In line with the positive effects of younger age on the survival of NSCLC BM patients, the overall disease status exemplified by higher KPS values also showed an expected positive effect on OS as well as survival post-BM surgery. Intriguingly, when comparing the OS values, we found that the preoperative KPS did not show a significant effect (p = 0.173, **Figure 1G**), whereas a postoperative KPS >70% did show a significant OS benefit (median 9.4 vs. 30.4, p < 0.001). When comparing the impact of KPS on survival post-BM resection, we found that both pre- and postop KPS scores >70% showed significantly increased survival (**Figures 2K**–M). This finding is important because it signifies the effect of BM surgery on the course of the disease—a postoperative decrease in the KPS score significantly impacts the course of disease with an overall reduction of survival.

Over 80% of the brain metastatic lesions were classified as adenocarcinomas, followed by squamous cells, with similar numbers recently reported (12). The rate and dissemination of genetic mutations was inconsistent between primary tumors and matched BM lesions, as exemplified by the difference in mutations affecting *TP53* (13.64% in primary tumor vs. 25.00% in BM) or *KRAS* (13.64% in primary tumor vs. 16.30% in BM). Apart from the possibility of technological disparities (as analyses were, in some cases, conducted in separate centers), this disparity correlates with the recent reports of altered genetic mutations observed in whole exome sequencing between 86 primary lung cancers and their matched BM (23). Surprisingly, the rate of *EGFR*-mutated lung cancers in our cohort was 9.78%, while comparable studies (15) reported 22.2% and 13.6% (12), respectively. Furthermore, mutations in *KRAS* have been reported as the most common genetic mutations in NSCLC, with mutation rates of approximately 30% (17), yet they were surpassed by the rate of *TP53* mutations in our observations.

A significant limitation of this study is the lack of the availability of the mutational status in the majority (65.15%) of patient cases. This may partly be due to the length of the observational period starting in 2015, as the rate of molecular analyses has significantly increased in the past years and could also represent a lack of accessible patient information, as the molecular analyses in our center are, in many cases, initiated by the departments continuing the treatment after neurosurgical resection (oncology and radiotherapy) and might not be readily accessible. Comparable studies (12) similarly reported the mutational status in 37.7% of cases. Nevertheless, a trend in survival benefit after neurosurgical resection was revealed in patients receiving ICB + CTX combination treatment and median OS reached 36.1 months in this group. Additional additional analyses of this subcohort will be further addressed in our future studies. Another limitation of our study is the lack of consistent follow-up information after patient discharge. Despite our efforts to incorporate every piece of available data, in our university hospital setting, patients are discharged early after neurosurgical intervention and the majority of postoperative radiotherapy and oncologic treatments are continued in ambulatory settings. The data from these institutions are not routinely made available to us.

As opposed to a study by Spanberger et al. (24), who described a significant survival benefit in patients with smaller peritumoral brain edema, in our cohort, we did not identify peritumoral brain edema as a significant factor on OS and survival after neurosurgical intervention. However, this may be, in part, due to the difference in the measurement and grading of PTB as well as interobserver bias. A recent study by Berghoff and colleagues showed a positive correlation between the extent of peritumoral brain edema and the density of CD8^+^ tumor-infiltrating lymphocytes (TILs) associated with favorable median OS times. However, one might argue that the increase in OS in this study cohort was mainly driven by the number of TILs rather than the extent of edema, as outlined in the significant correlation between the survival prognosis and the immunoscore (25). Importantly, this study analyzed BM from multiple primary tumors with the highest TIL infiltration observed in melanoma and renal cancer.

Taken together, our study highlights the importance of understanding the clinical course in NSCLC patients with BM for risk stratification and clinical decision-making in the era of interdisciplinary tumor boards. With improved surgical techniques and the introduction of intraoperative neuromonitoring or neuronavigation, the overall morbidity of BM resection has decreased over the past decades (10). Together with significant advances in targeted- and immuno-oncological treatment options, as well as improved radiotherapy protocols, patients diagnosed with NSCLC BM represent a patient population whose survival may significantly benefit from the use of aggressive multimodal therapy, even in the cerebrally metastatic—and especially so in the oligometastatic - stage.

## Methods

### Patient characteristics and study cohort

The electronic patient database was queried for patients aged 18 years or older who underwent surgery in our institution for NSCLC BM during the period 01/2014–12/2020. Key demographic and clinical parameters were identified, and the course of disease as well as follow-up screenings were extracted from the external physician’s letters where appropriate. The disease stage at the initial diagnosis was stratified according to the 7th edition of the UICC TNM classification. The smoking status was stratified according to the packages of cigarettes or equivalent tobacco products per day and years smoked (pack years, py).

Histological results were obtained from biopsies and surgically resected tumor tissues and examined regularly by the senior physicians of the departments of pathology and neuropathology at the University Medical Center Hamburg-Eppendorf. Patients with differing histological diagnoses were excluded from analysis. The mutational analyses and PD-L1 expression of primary tumor tissues were conducted in the department of pathology or extracted from external reports.

The period between the primary tumor diagnosis and BM was calculated from the date of the histological diagnosis of the primary tumor (either in our institution or from the external physician’s letters) until the date of the histological diagnosis of BM. OS was calculated from the time of the histological diagnosis of the primary NSCLC tumor or the histological diagnosis of BM to the date of death or last follow-up, extracted from the external physician’s letters where applicable.

A team of experienced neurosurgeons performed all surgeries and intraoperative navigation. Additional supportive techniques (i.e., neuromonitoring) were applied in the cases deemed necessary by the primary surgeon. Postoperative treatment decisions as well as decisions about follow-up screenings and procedures were reached within an interdisciplinary institutional tumor board, involving board-certified neurosurgeons, medical oncologists, radiation oncologists, and neuroradiologists.

Data analysis was performed on anonymized data sets. The study was conducted in accordance with the ethical guidelines of the Helsinki Declaration and the Hamburger Hospital Act.

### MRI and volumetry

The size, number, and extent of intracranial tumors were assessed in three-dimensional reconstructions of coronal, axial, and sagittal planes and measured in cm^3^ using Brainlab software (Version 4.0.0.159, Brainlab AG, Munich, Germany) in presurgical MRI scans [pre- and postcontrast T1-weighted sequences, T2-weighted sequences, and/or fluid attenuated inversion recovery (FLAIR) sequences]. For this, the regions of interest (ROIs) were contoured semimanually around contrast-enhanced regions in each slice of T1-weighted MRI images and PTB was identified as obvious perifocal hyperintensity using the same method in T2-weighted or FLAIR images. The MEI was measured from the tumor border and calculated by dividing the size of the tumor in T1-weighted images and the size of edema in T2-weighted images. The localization of BM was stratified into 1) supra-/infratentorial, 2) main cerebral lobe affected (frontal, parietal, temporal, occipital, cerebellar, and other), 3) depth from the cortex (0 = in direct contact with dura mater cerebri, 1 = less than 1 cm below cortex, 2 = >1 cm below cortex). In the case of multiple intracerebral lesions, the MEI and localization of the largest lesion were used for survival stratification.

### Statistical analysis

Statistical analysis was performed using SPSS Statistics Version 25 (IBM, Armonk, NY, USA). Metric data are presented with means and standard deviations (**Table 1**). Kaplan–Meier estimates were used as a non-parametric statistic to calculate survivals depending on patient characteristics (**Figures 1** and **2**; **Table 2**). The survival distributions were compared using the log-rank test. Median survival times, 95% confidence intervals, and patients at risk were provided for Kaplan–Meier estimates. Subsequently, significant patient characteristics were tested for multicollinearity using a Pearson correlation matrix and variance inflation factors. For Cox regression analysis (**Table 2**), significant patient characteristics were selected according to the results of collinearity analysis. Survival curves were calculated and plotted from Cox proportional hazards (**Figure 3**). Additionally, hazard ratios and the corresponding 95% confidence intervals are provided. Patients lost to follow-up or still alive at the end of the observation period were censored in statistical survival analysis. P-values lower than 0.05 were considered statistically significant and stratified as p < 0.05 (*), p < 0.01 (**), and p < 0.001 (***). All statistical analyses were reviewed by an experienced statistician from the Institute of Medical Biometry and Epidemiology, University Medical Center Hamburg Eppendorf.

## Data availability statement

The anonymized data sets are available on reasonable request. Requests to access the datasets should be directed to a.piffko@uke.de.

## Ethics statement

The studies involving human participants were reviewed and approved by Hamburg ethics committee. Written informed consent for participation was not required for this study in accordance with the national legislation and the institutional requirements.

## Author contributions

Conceptualization: AP, TS, and MM. Methodology: AP and TS. Software: AP and TS. Data curation: AP, BA, and IR. Formal analysis: AP, TS, and MM. Investigation: AP, BA, IR, and CM. Resources: LD, MW, KL, HW, and JS. Writing—original draft preparation: AP, MM, and TS. Writing—review and editing: CM, LD, HW, IR, and JS. Visualization: AP and TS. Funding acquisition: MW, KL, HW, and JS. All authors contributed to the article and approved the submitted version.

## Funding

The work was supported by the Bender Stiftung, Sander Stiftung (to HW and MM), and the Erich and Gertrud Roggenbuck Stiftung (to MM).

## Acknowledgments

The authors would like to thank Linda Krause, PhD, from the Institute of Medical Biometry and Epidemiology, University Medical Center Hamburg-Eppendorf for her close revision and support for this work.

## Conflict of interest

The authors declare that the research was conducted in the absence of any commercial or financial relationships that could be construed as a potential conflict of interest.

## Publisher’s note

All claims expressed in this article are solely those of the authors and do not necessarily represent those of their affiliated organizations, or those of the publisher, the editors and the reviewers. Any product that may be evaluated in this article, or claim that may be made by its manufacturer, is not guaranteed or endorsed by the publisher.

## Supplementary material

Supplementary Table 1 Patient no. at risk (including censored events) for 0, 50, 100, 150 and 200 months after BM diagnosis for

Supplementary Table 2 Patient no. at risk (including censored events) for 0, 20, 40, 60, 80, 100 and 120 months after BM surgery for **Figure 2**.
