# Supplementary material for: Clinical determinants impacting overall survival of patients with operable brain metastases from non-small cell lung cancer
Source: Front Oncol. 2022 Oct 20;12:951805. doi: 10.3389/fonc.2022.951805 (PMC9631813; doi:10.3389/fonc.2022.951805)
Supplement: Supplementary Table 1 — Patient no. at risk (including censored events) for 0, 50, 100, 150 and 200 months after BM diagnosis for [file Table_1.docx]

**Supplemental Table 1**

|  | | Patient no. at risk at month  (censored events) | | | | |
| --- | --- | --- | --- | --- | --- | --- |
|  |  | 0 | 50 | 100 | 150 | 200 |
| Fig. 1 a) | no | 97 | 14 (38) | 4 (46) | 1 (49) | 0 |
| Age >60 years | yes | 150 | 13 (37) | 3 (69) | 1 (70) | 0 |
| Figure 1 b) | male | 118 | 9 (51) | 1 (55) | 1 (56) | 0 |
| Gender | female | 133 | 19 (50) | 6 (62) | 1 (65) | 0 |
| Figure 1 c) | singular | 52 | 3 (25) | 1 (27) | 0 | 0 |
| Singular vs. solitary | solitary | 68 | 11 (32) | 1 (40) | 0 | 0 |
| Figure 1 d) | supratentorial | 176 | 18 (82) | 2 (91) | 0 | 0 |
| Localization of BM | infratentorial | 41 | 6 (14) | 1 (18) | 0 | 0 |
| Figure 1 e) | >2 | 66 | 2 (31) | 1 (32) | 0 | 0 |
| Number of BM | <2 | 153 | 22 (62) | 3 (76) | 1 (77) | 0 |
| Figure 1 f) | <1 | 109 | 11 (44) | 3 (50) | 1 (52) | 0 |
| Mass Edema Index | >1 | 21 | 1 (6) | 0 | 0 | 0 |
| Figure 1 g) | <70% | 61 | 6 (27) | 2 (30) | 1 (30) | 0 |
| KPS before BM surgery | >70% | 180 | 18 (69) | 4 (80) | 1 (83) | 0 |
| Figure 1 h) | <70% | 41 | 3 (17) | 0 | 0 | 0 |
| KPS after BM surgery | >70% | 195 | 21 (78) | 6 (90) | 2 (93) | 0 |
