# Supplementary material for: Clinical determinants impacting overall survival of patients with operable brain metastases from non-small cell lung cancer
Source: Front Oncol. 2022 Oct 20;12:951805. doi: 10.3389/fonc.2022.951805 (PMC9631813; doi:10.3389/fonc.2022.951805)
Supplement: Supplementary Table 2 — Patient no. at risk (including censored events) for 0, 20, 40, 60, 80, 100 and 120 months after BM surgery for Figure 2 . [file Table_2.docx]

**Supplemental Table 2**

|  | | Patient no. at risk at month (censored events) | | | | | | |
| --- | --- | --- | --- | --- | --- | --- | --- | --- |
|  |  | 0 | 20 | 40 | 60 | 80 | 100 | 120 |
| Fig. 2 a) | no | 98 | 28 (38) | 7 (38) | 2 (41) | 0 | 0 | 0 |
| Age >60 years | yes | 156 | 24 (57) | 4 (70) | 1 (72) | 1 (72) | 1 (72) | 1 (72) |
| Figure 2 b) | male | 118 | 18 (42) | 4 (51) | 2 (53) | 0 | 0 | 0 |
| Gender | female | 137 | 35 (11) | 9 (40) | 3 (64) | 1 (66) | 1 (66) | 1 (66) |
| Figure 2 c) | at diagnosis | 136 | 26 (37) | 6 (50) | 3 (53) | 1 (55) | 1 (55) | 1 (55) |
| Time to brain metastasis | >2 months | 107 | 24 (35) | 5 (49) | 1 (52) | 0 | 0 | 0 |
| Figure 2 d) | >2 | 68 | 9 (29) | 3 (32) | 1 (32) | 1 (32) | 1 (32) | 1 (32) |
| Number of BM | <2 | 157 | 40 (49) | 8 (72) | 2 (77) | 0 | 0 | 0 |
| Figure 2 e) | <7 cm^3^ | 70 | 20 (19) | 3 (31) | 0 | 0 | 0 | 0 |
| Size of BM | >7 cm^3^ | 64 | 11 (28) | 3 (24) | 2 (35) | 1 (36) | 1 (36) | 1 (36) |
| Figure 2 f) | singular | 53 | 5 (26) | 1 (29) | 1 (29) | 1 (29) | 1 (29) | 1 (29) |
| Singular vs. Solitary vs. multiple | solitary | 69 | 26 (18) | 6 (34) | 2 (37) | 0 | 0 | 0 |
|  | multiple | 64 | 8 (28) | 2 (31) | 1 (32) | 0 | 0 | 0 |
| Figure 2 g) | supratentorial | 173 | 38 (58) | 7 (81) | 2 (86) | 0 | 0 | 0 |
| Localization | infratentorial | 43 | 10 (14) | 4 (17) | 2 (18) | 1 (19) | 1 (19) | 1 (19) |
| Figure 2 h) | <1 | 111 | 26 (41) | 5 (47) | 1 (51) | 0 | 0 | 0 |
| Mass edema index | >1 | 21 | 5 (5) | 1 (7) | 1 (7) | 1 (7) | 1 (7) | 1 (7) |
| Figure 2 i) | at diagnosis | 147 | 29 (48) | 7 (63) | 3 (67) | 1 (69) | 1 (69) | 0 |
| Time of brain metastasis | after CT | 86 | 19 (24) | 4 (25) | 1 (37) | 0 | 0 | 0 |
|  | after IT | 20 | 4 (11) | 1 (13) | 0 | 0 | 0 | 0 |
| Figure 2 j) | <70% | 63 | 5 (29) | 1 (31) | 0 | 0 | 0 | 0 |
| KPS before BM surgery | >70% | 183 | 45 (50) | 11 (74) | 4 (80) | 1 (83) | 1 (83) | 1 (83) |
| Figure 2 k) | <70% | 43 | 4 (20) | 2 (20) | 1 (21) | 1 (21) | 1 (21) | 1 (21) |
| KPS after BM surgery | >70% | 198 | 46 (58) | 10 (74) | 3 (90) | 0 | 0 | 0 |
| Figure 2 l) | worse | 25 | 3 (7) | 2 (7) | 1 (17) | 1 (17) | 1 (17) | 1 (17) |
| KPS change by surgery | equal or better | 216 | 47 (71) | 10 (97) | 3 (103) | 0 | 0 | 0 |
